# Establishment and Verification of Prognostic Nomograms for Young Women With Breast Cancer Bone Metastasis

**DOI:** 10.3389/fmed.2022.840024

**Published:** 2022-04-12

**Authors:** Zhan Wang, Haiyu Shao, Qiang Xu, Yongguang Wang, Yaojing Ma, Diarra Mohamed Diaty, Jiahao Zhang, Zhaoming Ye

**Affiliations:** ^1^Department of Orthopedic Surgery, The Second Affiliated Hospital, Zhejiang University School of Medicine, Hangzhou, China; ^2^Orthopedics Research Institute of Zhejiang University, Hangzhou, China; ^3^Key Laboratory of Motor System Disease Research and Precision Therapy of Zhejiang Province, Hangzhou, China; ^4^Department of Orthopedics, Zhejiang Provincial People’s Hospital (Affiliated People’s Hospital, Hangzhou Medical College), Hangzhou, China; ^5^Department of Orthopaedics, Xuzhou Central Hospital, Xuzhou, China

**Keywords:** young women, breast cancer, bone metastasis, nomogram, survival, predictor

## Abstract

**Purpose:**

The prognosis of patients with metastatic breast cancer usually varies greatly among individuals. At present, the application of nomogram is very popular in metastatic tumors. The present study was conducted to identify independent survival predictors and construct nomograms among young women with breast cancer bone metastasis (BCBM).

**Patients and Methods:**

We searched the Surveillance, Epidemiology, and End Results (SEER) database to identify young women diagnosed with BCBM between 2010 and 2016. We first analyzed the potential risk factors of overall survival (OS) and cancer-specific survival (CSS) by applying univariate Cox regression analysis. Then we conducted multivariate Cox analysis to identify independent survival predictors. Based on significant independent predictors, we developed and validated novel prognostic nomograms by using the R version 4.1.0 software.

**Results:**

We finally identified 715 eligible young women with BCBM for survival analysis, of which 358 patients were in the training set, and 357 patients in the validation set. Approximately four-fifths of patients are between 31 and 40 years old. The 5-year OS and CSS rates of this research population were 41.9 and 43.3%, respectively. Multivariate analysis revealed seven independent predictors of both OS and CSS, including race, tumor subtype, tumor size, surgical treatment, brain metastasis, liver metastasis, and lung metastasis. Based on these predictors, we developed and validated OS and CSS nomograms. The C-index of the OS nomogram reached 0.728 and 0.73 in the training and validation sets, respectively. The C-index of the CSS nomogram reached 0.743 and 0.695 in the training and validation sets, respectively. Meanwhile, high quality calibration plots were revealed in both OS and CSS nomograms.

**Conclusion:**

The current novel nomograms can provide an individualized survival evaluation of young women with BCBM and instruct clinicians to treat them appropriately.

## Introduction

Although female breast cancer (BC) under the age of 40 accounts for about 7–10% of all BCs (1), it is the most common malignant tumor among young patients (2). Moreover, young BC is the leading cause of cancer death in this age group (3). Previous studies have shown that young BC patients present with more aggressive clinical characteristics and poor outcome compared with elderly (2, 4, 5). However, young metastatic BC patients might have better prognosis than elderly patients (6). BC most often metastasizes to the bone, which not only negatively affects the quality of life of patients, but also affects the longevity of patients (7). Although the diagnosis and treatment of BC has made great progress, bone metastasis is still a significant challenge for clinicians. Currently, there are limited studies on the clinical features and prognosis of young women with breast cancer bone metastasis (BCBM).

Currently, driven by the clinical needs, more and more researchers focus on establishing clinical models for predicting the outcome and guiding the clinical management (8, 9). Nomogram as a popular quantitative predictive model, has been successfully applied to calculate and predict the survival of cancer patients (10, 11). More importantly, nomograms can ensure the accuracy of prognostic prediction and visually display patients’ prognosis prediction results. To the best of our knowledge, there are limited studies on the clinical characteristics and survival prediction of women patients with BCBM under age 40. This is the first presentation of developing and validating novel nomograms of young women patients with BCBM by using the Surveillance, Epidemiology, and End Results (SEER) database.

## Materials and Methods

### Patients Selection

Clinical data from the SEER database on young BCBM were obtained by using the case-listing session on the SEER*Stat version 8.3.9 software. We selected the primary tumor sites of BC by using the Site recode ICD-O-3/WHO 2008 “Breast.” Meanwhile, we set the Age at diagnosis to be ≤40 years and SEER Combined Mets at DX-bone (2010+) to be YES. As a result, we obtained clinical information on all young BC patients with bone metastasis. Male patients and patients with other unknown variables were excluded. This study collected patients’ information from a public database and did not contain any patient-identified information. Thus, this study was exempted from medical ethics review.

We obtained data from the SEER database, including race, age at diagnosis, gender, laterality, tumor grade, histological type, tumor subtype, tumor size, surgery, radiotherapy, chemotherapy, visceral metastasis, vital status, survival time, and cause of death. The “young breast cancer” was defined as women aged under 40 years. Surgery or radiotherapy in the present study refers to treatment for breast cancer. According to previous studies (12, 13), overall survival (OS) and cancer-specific survival (CSS) were defined as the time from diagnosis to the time of death attributed to any cause and breast cancer, respectively. Both OS and CSS were used as endpoints of the present study.

All eligible patients were randomly divided into two groups in a 1:1 ratio, which consisted of the training set and validation set. We used the training set for constructing nomograms and the validation set for validating nomograms.

### Statistical Analysis

Univariate Cox regression analysis was first performed to analyze potential risk factors. Then we conducted multivariate Cox regression analysis to identify independent survival predictors. Meanwhile, hazard ratio (HR) and their 95% confidence interval (95% CIs) were presented in both univariate and multivariate analysis. Two-sided *p* value less than 0.05 was considered of significance. The above statistical analyses were carried out using the SPSS 23.0 software.

Nomograms were constructed and validated by using the R version 4.1.0 software^1^ through comprehensive analysis of all independent predictors. We used the concordance index (C-index) and calibration plots to assess the prognostic performance of the nomograms.

To achieve this purpose, bootstraps with 1,000 resamples were performed in both training and validation sets.

## Results

### Clinicopathologic Characteristics

Ultimately, we identified 715 eligible young patients with BCBM from 2010 to 2016 in SEER, which consisted of 358 patients in the training set, and 357 patients in the validation set. The clinicopathologic characteristics of this special population were summarized in Table 1. White cases accounted for approximately 70%. Most patients were found in the 31-40 years age group (82.5%), with 20–30 years age group accounting for 17.5%. The distribution ratio of left and right tumor sites was similar. 47.5% of all cases were pathologically graded as Grade I or II, and 54.3% were graded as Grade III or IV. The most common histological subtype was infiltrating duct carcinoma, NOS (84.3%). More than half of patients (53.0%) were Luminal A. Of this study population, 44.8% received surgery, 47.6% received radiotherapy, and 83.6% received chemotherapy. The proportions of these patients with brain metastasis, liver metastasis and lung metastasis were 5.2, 29.9, and 18.3%, respectively. It should be noted that the majority of patients (82.2%) had lymph node metastasis. The 3- and 5-year OS rates were 61.5 and 41.9%, respectively. The 3- and 5-year CSS rates were 62.6 and 43.3%, respectively.

**TABLE 1:** Demographics of 715 young breast cancer bone metastasis.

Variable	Value
**Race**	
White	496(69.4%)
Black	142(19.9%)
Others	77(10.8%)
**Age(years)**	
20–30	125(17.5%)
31–40	590(82.5%)
**Laterality**	
Left	358(50.1%)
Right	357(49.9%)
**Tumor grade**	
Low grade (I/II)	327(45.7%)
High grade (III/IV)	388(54.3%)
**Histological type**	
Infiltrating duct carcinoma, NOS	603(84.3%)
Others	112(15.7%)
**Tumor subtype**	
Luminal A	379(53.0%)
Luminal B	202(28.3%)
HER2+	67(9.4%)
Triple-negative	67(9.4%)
**Tumor size (cm)**	
<3	209(29.2%)
3–6	294(41.1%)
>6	212(29.7%)
**Surgery**	
Yes	320(44.8%)
No	395(55.2%)
**Radiotherapy**	
Yes	340(47.6%)
No	375(52.4%)
**Chemotherapy**	
Yes	598(83.6%)
No	117(16.4%)
**Brain metastasis**	
No	678(94.8%)
Yes	37(5.2%)
**Liver metastasis**	
No	501(70.1%)
Yes	214(29.9%)
**Lung metastasis**	
No	584(81.7%)
Yes	131(18.3%)
**Lymph node metastasis**	
No	127(17.8%)
Yes	588(82.2%)
**Dead**	
Yes	268(37.5%)
No	447(62.5%)
3-year OS rate	61.50%
3-year CSS rate	62.60%
5-year OS rate	41.90%
5-year CSS rate	43.30%

*OS, overall survival, CSS, cancer-specific survival.*

### Cox Regression Analyses

As shown in Table 2, nine significant variables of OS were identified by using univariate Cox regression analysis, including race, tumor grade, tumor subtype, tumor size, surgery, radiotherapy, brain metastasis, liver metastasis, and lung metastasis. Race, tumor grade, tumor subtype, tumor size, surgery, brain metastasis, liver metastasis, and lung metastasis were shown to be significantly associated with CSS. Only significant survival predictors generated in univariate analysis were included in multivariate analysis. The detailed results of the multivariate analysis from the training set were summarized in Table 3. Seven independent predictors of both OS and CSS were identified, including race, tumor subtype, tumor size, surgery, brain metastasis, liver metastasis, and lung metastasis.

**TABLE 2:** Univariate Cox analysis of survival in the training set.

Variable	OS		CSS	
	HR (95% CI)	*P*	HR (95% CI)	*P*
**Race**				
White	1		1	
Black	2.026 (1.391–2.952)	< 0.001	1.976 (1.343–2.907)	0.001
Others	0.850 (0.463–1.559)	0.599	1.141 (0.661–1.969)	0.636
**Age (years)**				
20–30	1		1	
31–40	1.025 (0.667–1.575)	0.911	1.033 (0.655–1.632)	0.888
**Laterality**				
Left	1		1	
Right	1.011 (0.720–1.419)	0.949	0.899 (0.648–1.247)	0.524
**Tumor grade**				
Low grade (I/II)	1		1	
High grade (III/IV)	1.6 (1.125–2.275)	0.009	1.394 (1.000–1.944)	0.05
**Histological type**				
Infiltrating duct carcinoma, NOS	1		1	
Others	1.247 (0.818–1.903)	0.305	0.849 (0.529–1.363)	0.498
**Tumor subtype**				
Luminal A	1		1	
Luminal B	0.670 (0.421–1.067)	0.092	0.711 (0.457–1.105)	0.13
HER2+	0.995 (0.569–1.739)	0.985	1.130 (0.658–1.942)	0.657
Triple-negative	5.724 (3.533–9.272)	< 0.001	6.425 (3.983–10.365)	< 0.001
**Tumor size (cm)**				
<3	1		1	
3–6	1.752 (1.142–2.686)	0.01	1.752 (1.142–2.686)	0.01
>6	1.887 (1.215–2.931)	0.005	1.887 (1.215–2.931)	0.005
**Surgery**				
Yes	1		1	
No	2.510 (1.752–3.598)	< 0.001	2.137 (1.514–3.017)	< 0.001
**Radiotherapy**				
Yes	1		1	
No	1.568 (1.109–2.217)	0.011	1.263 (0.908–1.757)	0.165
**Chemotherapy**				
Yes	1		1	
No	1.203 (0.783–1.847)	0.399	1.276 (0.848–1.920)	0.243
**Brain metastasis**				
No	1		1	
Yes	3.235 (1.737–6.023)	< 0.001	7.323 (4.067–13.185)	< 0.001
**Liver metastasis**				
No	1		1	
Yes	2.174 (1.533–3.083)	< 0.001	2.089 (1.494–2.920)	< 0.001
**Lung metastasis**				
No	1		1	
Yes	2.658 (1.791–3.943)	< 0.001	2.8 (1.945–4.032)	< 0.001
**Lymph node metastasis**				
No	1		1	
Yes	0.907 (0.594–1.384)	0.65	1.167 (0.758–1.797)	0.484

**TABLE 3:** Multivariate Cox regression analysis of survival in the training set.

Variable	OS		CSS	
	HR (95% CI)	*P*	HR (95% CI)	*P*
**Race**				
White	1		1	
Black	1.683 (1.135–2.497)	0.01	1.654 (1.106–2.476)	0.014
Others	0.966 (0.510–1.830)	0.916	1.296(0.731–2.296)	0.375
**Tumor grade**				
Low grade (I/II)	1		1	
High grade (III/IV)	1.335 (0.882–2.021)	0.172	1.138 (0.771–1.679)	0.515
**Tumor subtype**				
Luminal A	1		1	
Luminal B	0.553 (0.337–0.907)	0.019	0.517 (0.323–0.827)	0.006
HER2+	0.627 (0.343–1.146)	0.13	0.748 (0.423–1.324)	0.319
Triple-negative	3.774 (2.153–6.615)	< 0.001	5.349 (3.129–9.144)	< 0.001
**Tumor size (cm)**				
<3	1		1	
3–6	1.100 (0.711–1.704)	0.668	1.943 (1.245–3.034)	0.003
>6	1.721 (1.070–2.766)	0.025	2.203 (1.378–3.522)	0.001
**Surgery**				
Yes	1		1	
No	1.941 (1.302–2.893)	0.001	1.882 (1.295–2.735)	0.001
**Radiotherapy**				
Yes	1		–	
No	1.196 (0.815–1.754)	0.36	–	−⁣−
**Brain metastasis**				
No	1		1	
Yes	3.015 (1.464–6.209)	0.003	4.028 (2.021–8.028)	< 0.001
**Liver metastasis**				
No	1		1	
Yes	1.540 (1.043–2.275)	0.03	1.528 (1.039–2.247)	0.031
**Lung metastasis**				
No	1		1	
Yes	1.748 (1.124–2.718)	0.013	2.214 (1.459–3.359)	< 0.001

### Nomogram Construction and Validation

**FIGURE 1:**
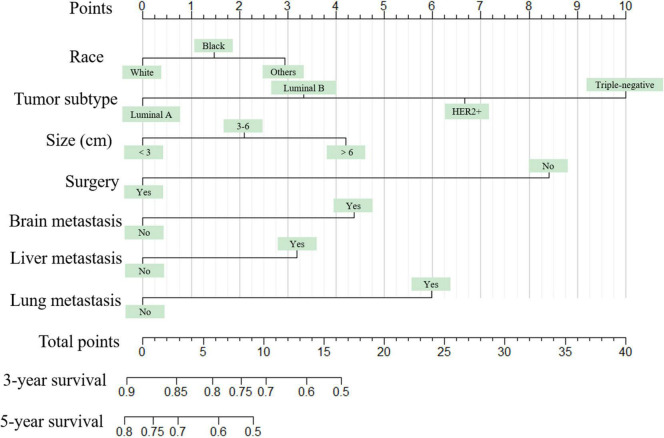
The graph shows the nomogram predicting 3- and 5-year overall survival of young women with breast cancer bone metastasis. The nomogram summed the points identified on the scale for each predictor. The total points projected on the bottom scales indicate the probabilities of 3- and 5-year overall survival.

**FIGURE 2:**
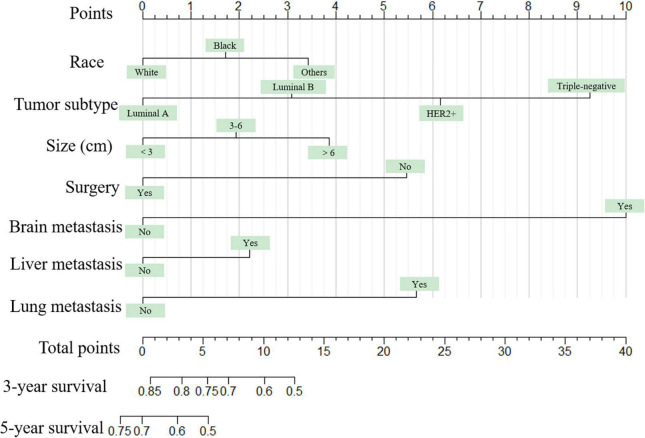
The graph shows the nomogram predicting 3- and 5-year cancer-specific survival of young women with breast cancer bone metastasis. The nomogram summed the points identified on the scale for each predictor. The total points projected on the bottom scales indicate the probabilities of 3- and 5-year overall survival.

**FIGURE 3:**
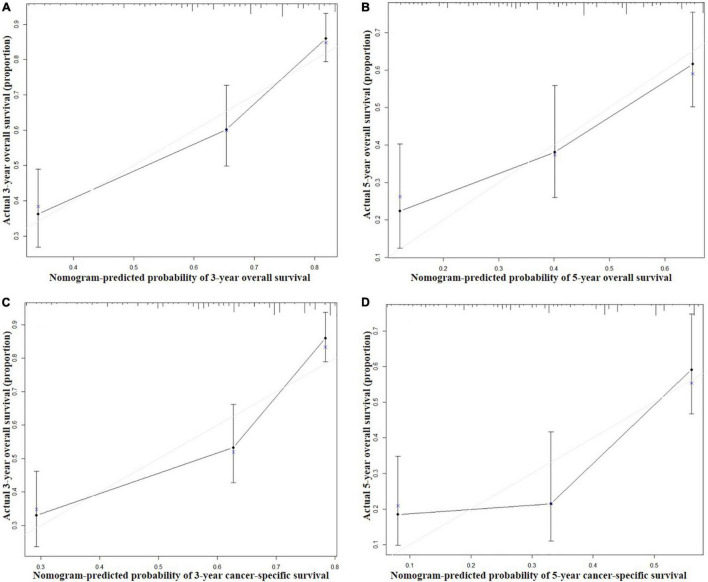
Calibration curves for 3-year **(A)** and 5-year **(B)** overall survival; and 3-year **(C)** and 5-year **(D)** cancer-specific survival in the training set. The X axis represents the nomogram predicted survival rate, whereas the Y axis represents the actual survival rate.

**FIGURE 4:**
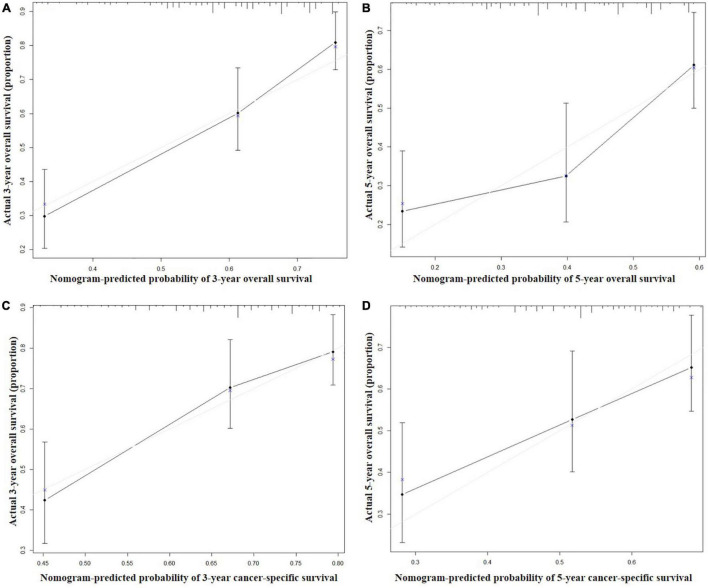
Calibration curves for 3-year **(A)** and 5-year **(B)** overall survival; and 3-year **(C)** and 5-year **(D)** cancer-specific survival in the validation set. The X axis represents the nomogram predicted survival rate, whereas the Y axis represents the actual survival rate.

Seven independent survival predictors revealed by multivariate analysis from the training set were incorporated in constructing nomograms of OS (Figure 1) and CSS prediction (Figure 2). The C-index of the OS nomogram reached 0.728 and 0.73 in the training and validation sets, respectively. The C-index of the CSS nomogram reached 0.743 and 0.695 in the training and validation sets, respectively. The above results showed that these models had better discrimination ability. Then we further validated performance of nomograms by using calibration plots. As shown in Figures 3, 4, calibration plots showed high consistency between observed and predicted survival.

The nomogram summed the scores identified on the scale for each predictor. The total points projected on the bottom scales indicate the 3-year and 5-year survival rates of each patient. The specific scores assigned to each predictor were summarized in Table 4.

**TABLE 4:** Point assignment for each variable included in the nomograms.

Variable	OS nomogram	CSS nomogram
*Race*		
White	0	0
Black	1.5	1.7
Others	2.9	3.4
*Tumor subtype*		
Luminal A	0	0
Luminal B	3.3	3.1
HER2+	6.7	6.2
Triple-negative	10	9.3
*Tumor size (cm)*		
<3	0	0
3–6	2.1	1.9
>6	4.2	3.9
*Surgery*		
Yes	0	0
No	8.42	5.5
*Brain metastasis*		
No	0	0
Yes	4.4	10
*Liver metastasis*		
No	0	0
Yes	3.2	2.2
*Lung metastasis*		
No	0	0
Yes	6	5.7

## Discussion

Although the diagnosis and treatment of BC has made great progress, the occurrence of bone metastasis brings great challenges to clinicians. Young patients diagnosed as BCBM are a rare group, and insufficient attention has been paid to them in the past. In the present study, we first defined the clinicopathological features and prognosis of this special population. More importantly, we developed an insightful and applicable tool to accurately predict the survival of them.

Young BCBM and overall BCBM have multiple similar clinicopathologic characteristics, such as distribution of race, laterality, tumor grade, histological type, tumor subtype (12, 14, 15). Interestingly, the prognosis of BC in young women is usually poor, but for BC patients who developed bone metastasis, young women had a survival advantage. Our study showed the 5-year OS and CSS rates were 41.9 and 43.3%, respectively, which was higher than those of previous studies on BCBM (<34%) (12, 15). Notably, the proportion of young women with BCBM receiving various treatments (surgery, 44.8%; radiotherapy, 47.6%; chemotherapy, 83.6%) was higher than that of the overall BCBM patients (surgery, 36.4%; radiotherapy, 42.4%; chemotherapy, 62.3%) (12), indicating that they were more inclined to receive active therapies.

In the current study, we confirmed seven independent risk factors of both OS and CSS, including race, tumor subtype, tumor size, surgery, brain metastasis, liver metastasis, and lung metastasis, through both univariate analysis and multivariate Cox analysis. Based on these independent survival predictors, we constructed reliable graphical representation models to predict the survival of each young women with BCBM. To our knowledge, this was the first study to establish nomograms for predicting the prognosis of young patients with BCBM, which was of great significance in decision-making given the long survivorship period.

In recent years, the clinical application of nomograms has gradually increased, not only for predicting the prognosis of tumors, but also for predicting the outcome of non-tumor diseases (16–18). Many previous studies indicated that cancer nomograms with C-index values ranging from 0.6 to 0.7, revealed good prognostic accuracy and clinical applicability (19, 20). The minimum C value of the nomogram in this study was 0.695, and the other three C values were all greater than 0.7, which indicated a better prognostic accuracy. In addition, the calibration curves showed the predicted probabilities produced by the nomograms in the training and validation sets were compared with the actual probabilities.

There are some shortcomings of this study. First, the study was a retrospective study based on SEER database, which has certain bias. Second, we cannot obtain all prognostic factors comprehensively from the SEER database. Third, due to the rarity of young BCBM, external validation was not conducted to further evaluate our nomograms. Even though nomogram-based clinical modeling is quite user-friendly, which can accurately calculate and predict the survival rate of each patient.

## Conclusion

Our nomograms provide an effective approach for clinicians to assess the prognosis of young BCBM individually. However, further validation by multicenter clinical trials is needed to refine our models and promote its clinical application.

## Data Availability Statement

The raw data supporting the conclusions of this article will be made available by the authors, without undue reservation.

## Ethics Statement

Ethical review and approval was not required for the study on human participants in accordance with the local legislation and institutional requirements. Written informed consent for participation was not required for this study in accordance with the national legislation and the institutional requirements.

## Author Contributions

ZW and ZY conceived and designed the study. ZW, HS, and QX collected the data. ZW, HS, QX, YW, YM, DD, and JZ performed the statistical analysis. ZW wrote the manuscript. HS, QX, YW, YM, and ZY revised the manuscript. All authors read and approved the final manuscript.

## Conflict of Interest

The authors declare that the research was conducted in the absence of any commercial or financial relationships that could be construed as a potential conflict of interest.

## Publisher’s Note

All claims expressed in this article are solely those of the authors and do not necessarily represent those of their affiliated organizations, or those of the publisher, the editors and the reviewers. Any product that may be evaluated in this article, or claim that may be made by its manufacturer, is not guaranteed or endorsed by the publisher.

## Footnotes

1
https://www.r-project.org/

## References

[1] Rossi L Mazzara C Pagani O. Diagnosis and treatment of breast cancer in young women. *Curr Treat Options Oncol.* (2019) 20:86. 10.1007/s11864-019-0685-7 31776799

[2] Hu X Myers KS Oluyemi ET Philip M Azizi A Ambinder EB. Presentation and characteristics of breast cancer in young women under age 40. *Breast Cancer Res Treat.* (2021) 186:209–17. 10.1007/s10549-020-06000-x 33136248

[3] Eiriz IF Vaz Batista M Cruz Tomás T Neves MT Guerra-Pereira N Braga S. Breast cancer in very young women-a multicenter 10-year experience. *ESMO Open.* (2021) 6:100029. 10.1016/j.esmoop.2020.100029 33399090 PMC7807935

[4] Shoemaker ML White MC Wu M Weir HK Romieu I. Differences in breast cancer incidence among young women aged 20-49 years by stage and tumor characteristics, age, race, and ethnicity, 2004-2013. *Breast Cancer Res Treat.* (2018) 169:595–606. 10.1007/s10549-018-4699-9 29445940 PMC5955792

[5] Slaoui M Mouh FZ Ghanname I Razine R El Mzibri M Amrani M. Outcome of breast cancer in moroccan young women correlated to clinic-pathological features, risk factors and treatment: a comparative study of 716 cases in a single institution. *PLoS One.* (2016) 11:e0164841. 10.1371/journal.pone.0164841 27760178 PMC5070817

[6] Liu W Xiong XF Mo YZ Chen WG Li M Liang R Young age at diagnosis is associated with better prognosis in stage IV breast cancer. *Aging.* (2019) 11:11382–90. 10.18632/aging.102536 31829978 PMC6932875

[7] Venetis K Piciotti R Sajjadi E Invernizzi M Morganti S Criscitiello C Breast cancer with bone metastasis: molecular insights and clinical management. *Cells.* (2021) 10:1377. 10.3390/cells10061377 34199522 PMC8229615

[8] Li W Xiao Y Xu X Zhang Y. A novel nomogram and risk classification system predicting the cancer-specific mortality of patients with initially diagnosed metastatic cutaneous melanoma. *Ann Surg Oncol.* (2021) 28:3490–500. 10.1245/s10434-020-09341-5 33191484

[9] Huang XT Huang CS Liu C Chen W Cai JP Cheng H Development and validation of a new nomogram for predicting clinically relevant postoperative pancreatic fistula after pancreatoduodenectomy. *World J Surg.* (2021) 45:261–9. 10.1007/s00268-020-05773-y 32901325

[10] Liu H Lv L Qu Y Zheng Z Zhao J Liu B Prediction of cancer-specific survival and overall survival in middle-aged and older patients with rectal adenocarcinoma using a nomogram model. *Transl Oncol.* (2021) 14:100938. 10.1016/j.tranon.2020.100938 33186890 PMC7658496

[11] Dai L Wang W Liu Q Xia T Wang Q Chen Q Development and validation of prognostic nomogram for lung cancer patients below the age of 45 years. *Bosn J Basic Med Sci.* (2021) 21:352–63. 10.17305/bjbms.2020.5079 33091332 PMC8112561

[12] Wang Z Cheng Y Chen S Shao H Chen X Wang Z Novel prognostic nomograms for female patients with breast cancer and bone metastasis at presentation. *Ann Transl Med.* (2020) 8:197. 10.21037/atm.2020.01.37 32309344 PMC7154431

[13] Wang Z Wu B Zhou Y Huang X Pan W Liu M Predictors of the survival of primary and secondary older osteosarcoma patients. *J Cancer.* (2019) 10:4614–22. 10.7150/jca.32627 31528225 PMC6746122

[14] Liu D Wu J Lin C Andriani L Ding S Shen K Breast subtypes and prognosis of breast cancer patients with initial bone metastasis: a population-based study. *Front Oncol.* (2020) 10:580112. 10.3389/fonc.2020.580112 33344236 PMC7739957

[15] Li X Zhang X Liu J Shen Y. Prognostic factors and survival according to tumour subtype in women presenting with breast cancer bone metastases at initial diagnosis: a SEER-based study. *BMC Cancer.* (2020) 20:1102. 10.1186/s12885-020-07593-8 33187507 PMC7666499

[16] Su Y Yuki M Hirayama K Otsuki M. Development and internal validation of a nomogram to predict post-stroke fatigue after discharge. *J Stroke Cerebrovasc.* (2021) 30:105484. 10.1016/j.jstrokecerebrovasdis.2020.105484 33253982

[17] Dong YM Sun J Li YX Chen Q Liu QQ Sun Z Development and validation of a nomogram for assessing survival in patients with COVID-19 pneumonia. *Clin Infect Dis.* (2021) 72:652–60. 10.1093/cid/ciaa963 32649738 PMC7454485

[18] Deng F Peng M Li J Chen Y Zhang B Zhao S. Nomogram to predict the risk of septic acute kidney injury in the first 24 h of admission: an analysis of intensive care unit data. *Renal Fail.* (2020) 42:428–36. 10.1080/0886022x.2020.1761832 32401139 PMC7269058

[19] Zuo Z Zhang G Song P Yang J Li S Zhong Z Survival nomogram for stage IB non-small-cell lung cancer patients, based on the SEER database and an external validation cohort. *Ann Surg Oncol.* (2021) 28:3941–50. 10.1245/s10434-020-09362-0 33249521

[20] Lin S Mo H Li Y Guan X Chen Y Wang Z Development and validation of a nomogram for predicting survival of advanced breast cancer patients in China. *Breast.* (2020) 53:172–80. 10.1016/j.breast.2020.08.004 32836201 PMC7451432

